# A Simple Metal-Free Cyclization for the Synthesis of 4-Methylene-3-Substituted Quinazolinone and Quinazolinthione Derivatives: Experiment and Theory

**DOI:** 10.3389/fchem.2019.00584

**Published:** 2019-08-16

**Authors:** Huihui Yan, Xu-Qiong Xiao, Robert C. Hider, Yongmin Ma

**Affiliations:** ^1^School of Pharmaceutical and Chemical Engineering, Taizhou University, Taizhou, China; ^2^School of Pharmaceutical Science, Zhejiang Chinese Medical University, Hangzhou, China; ^3^Key Laboratory of Organosilicon Chemistry and Material Technology of Ministry of Education, Hangzhou Normal University, Hangzhou, China; ^4^Institute of Pharmaceutical Science, King's College London, London, United Kingdom

**Keywords:** cyclization, quinazolinone, quinazolinthione, quantum chemical calculations, SMD-B3LYP

## Abstract

A new series of 3-substituted 4-methylene-quinazolinthiones and 4-methylene-quinazolinones were synthesized in moderate to excellent yield through a simple reaction of 2-aminoacetophenones with isocyanates or isothiocyanates. The reaction shows good tolerance of many important functional groups in the presence of air and water under metal-free conditions. Only water is produced as a coproduct, rendering this “green” methodology a highly versatile and eco-friendly alternative to the existing methods for the construction of the quinazolinone/quinazolinthione framework. We have interpreted the reaction mechanism by use of quantum chemical calculations on the basis of state-of-the-art computational methods SMD-B3LYP-D3(BJ)/BS1//B3LYP/BS1.

## Introduction

Quinazolinones and their derivatives occur frequently in natural and synthetic pharmaceutical products. Such compounds act as inhibitors of inducible and neuronal nitric oxide synthases (Camacho et al., 2016), possess anti-melanogenesis activity (Thanigaimalai et al., 2010), inhibit aldosterone synthase (CYP11B) and act as antagonists of platelet activating factor (Walser et al., 1991; Grombein et al., 2015). In recent years, many studies have been directed toward the design of 3,4-dihydroquinazolin-2(*1H*)-ones for the modulation of cardiotonic contractility (Campbell et al., 1988), inhibition of methionyl-tRNA synthetase (Buckner et al., 2015) and inhibition of HIV-1 Tat-TAR (Zeiger et al., 2014) together with the design of highly selective naked-eye sensors (Mei et al., 2012).

**Scheme 1:**
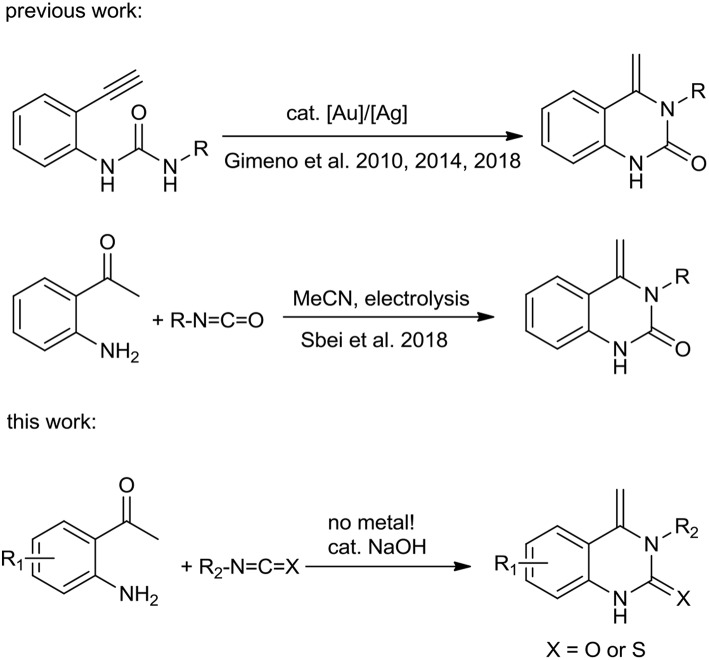
Previous and present work for the synthesis of 4-methylene-quinazolin-2-ones.

Many 3,4-dihydroquinazolin-2(1*H*)-one derivatives have been previously synthesized (Huang et al., 2008; Khan et al., 2014, 2015, 2016; Kshirsagar, 2015; Camacho et al., 2016; Maiden and Harrity, 2016; Awad et al., 2018; Zhang et al., 2018; Elkholy et al., 2019; Gatadi et al., 2019; Long et al., 2019; Wang et al., 2019). For example, Wang et al. (2009) reported the synthesis of 4-alkyl-2(1H)-quinazolinones *via* the cyclization of 1-(2-alkynyl-phenyl)ureas catalyzed by TfOH and Saunthwal et al. (2015) described a green and catalyst-free straightforward tandem synthesis of functionalized tetrahydroquinazolines from 2-aminophenylacrylate. Sawant et al. (2015) developed a microwave-promoted sequential cyclization-Mannich reaction of ketones, *o*-formyl carbamates and primary amines to form polyfunctionalized 3,4-dihydroquinazolinones. Fukamachi et al. (2010) described a selective construction of 3,4-dihydroquinazoline-2-thiones by reacting 3-(2-isothiocyanatophenyl)propanoic derivatives with primary amines. 3,4-Dihydroquinazoline-2-thiones can also be synthesized from isothiocyanates with 2-aminophenyl acrylates or 2-amino chalcones (Hua et al., 2014; Xie et al., 2016). However, there are very few reports on the production of 4-alkenylquinazolinones and 4-alkenylquinazolinthiones. The first 4-alkenylquinazolinone to be successfully synthesized starting from quinoline-1-carboxamides was achieved in the presence of strong acids (Brack, 1969). Molina et al. (1993) utilized iminophosphorane and 2 equivalents of isocyanate to form 4-methylen-4*H*-3,1-benzoxazine which upon heating undergoes elimination and rearrangement to furnish 3-substituted 4-methenylquinazolinones. There were only two pioneering methodologies for the synthesis of 4-methylen-3,4-dihydroquinazolin-2-ones, one from 1-(*o*-alkynylaryl)ureas catalyzed by an Au(I)-complex (Scheme 1, top) (Gimeno et al., 2010, 2014a,b) and the other by reaction of 2-aminoacetophenone with the cyanomethyl anion electrogenerated by acetonitrile reduction at a graphite electrode (Scheme 1, middle) (Sbei et al., 2018). However, these reactions suffer from one or more drawbacks, such as expensive catalysts/ligands, harsh reaction conditions and multi-step preparation of raw materials. Thus, simple and efficient approaches to 4-alkenylquinazolinons and 4-alkenylquinazolinthione synthesis from readily available starting materials remains an important target. Herein, we report the synthesis of 4-alkenylquinazolinones and 4-alkenylquinazolinthiones from readily available materials (Scheme 1, bottom). The process is simple, proceeds under mild conditions in the absence of a catalyst for the synthesis of 4-alkenylquinazolinthiones and with catalytic amounts of NaOH, for the synthesis of 4-alkenylquinazolinones.

## Results and Discussion

**Scheme 2:**
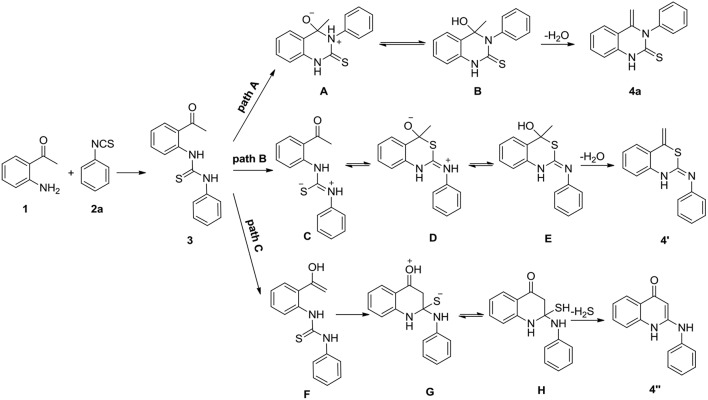
Possible main pathways for the addition/cyclization of compounds **1** and **2a**.

Nucleophilic addition of 2-aminoacetophenones **1** with isothiocyanates **2a** results in a formation of 1,3-diarylthiourea **3**. In principle, heterocyclization of compound **3** may occur by three different mechanisms (Scheme 2). Firstly, quinazolinthione **4a** can result from the nucleophilic attack of N-3 of compound **3** on the carbonyl group of the ketone followed by removal of water (path A). Secondly, compound **3** is transformed to its mesomeric form **C** and the latter cyclizes through an addition reaction to the carbonyl group of the ketone leading to the formation of benzothiazinimine **4′** by the elimination of one molecule of water (path B). Thirdly, compound **3** is first converted to an enol form and the enol of intermediate **F** attacks the thiocarbonyl group of thiourea followed by a loss of H_2_S to afford quinolinone **4′′** (path C).

**Figure 1:**
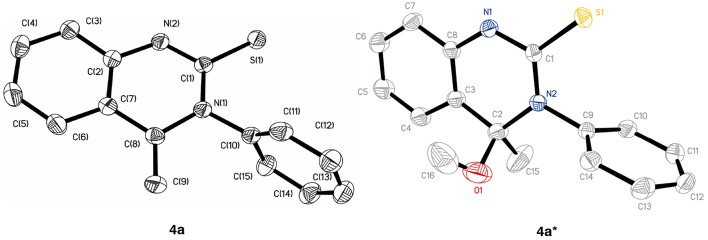
Single crystal X-ray structures of compound **4a** and its derivative 4a*.

In an initial experiment, the reaction of 2-aminoacetophenone **1** with phenyl isothiocyanate **2a** was undertaken in acetonitrile (MeCN) at room temperature. Not surprisingly, the main nucleophilic addition product **3** was isolated (yield: 50%). Apart from this, a small proportion of a new compound was detected (entry 1, Table 1). ^1^H NMR spectra demonstrated that there are two double peaks at 4.82 (one proton) and 3.69 ppm (one proton), respectively, ruling out the existence of product **4′′**. To identify the product structure as being either **4a** or **4′**, single crystals were isolated and the structure characterized by X-ray crystallographic analysis. It was established that the quinazolinthione **4a** was the correct structure (CCDC1906269, Figure 1, left, see Supporting Information for details). However, to our surprise, when crystals of the compound were incubated in methanol, an additional methoxy group was attached to 4-position of the quinazolinthione ring (CCDC1906268, Figure 1, right), indicating that the methylene group of compound **4a** is an active site and performs a further coupling reaction. This observation is currently under investigation.

**Table 1:** Optimization of reaction conditions^a^.

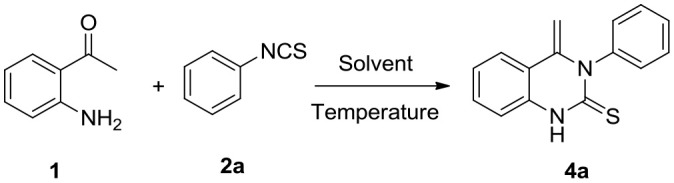			
**Entry**	**Solvent/T (°C)**	**Time (h)**	**Yield (%)**
1	MeCN/20	20	10
2	MeCN/50	12	58
3	EtOAc/50	24	45
**4**	**MeCN**/**reflux**	**1.5**	**96**
5	EtOAc/reflux	12	60
6	DCM/reflux	8	25
7	THF/reflux	5	39
8	Benzene/reflux	5	64
9	Dioxane/reflux	5	50

a *Reaction conditions: **1** (1 mmol) and **2a** (1.1 mmol) in a solvent (5 mL) at the indicated reaction conditions. Bold value indicates the best condition of the reaction*.

**Figure 2:**
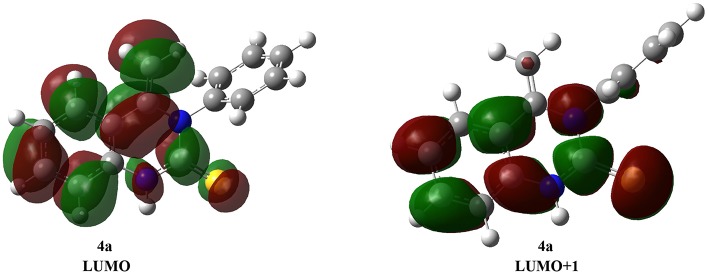
The optimized Structure of **4a** LUMO and LUMO+1 orbital.

Consistent with Fukui's theory, LUMO (the lowest unoccupied molecular orbital) and E_LUMO_ (the energy of LUMO) are clear, as is the extent of molecular susceptibility toward attack by external electrons (Dhami et al., 1997; Yan et al., 2019). Thus, the carbon-carbon double bond character of the **4a** structure is also represented by strong binding interactions in LUMO and LUMO+1 (Figure 2). This calculation provides a realistic description of the nucleophilic attack on the double bond on **4a**.

We optimized the reaction conditions in order to obtain the best yield by screening solvents and temperatures. As shown in Table 1, the yield was improved to 58% when the reaction was increased to 50°C (entry 2). In parallel, replacement of acetonitrile with ethyl acetate resulted in lower yield, even with an extended time. The reaction was found to occur with high efficiency at a short reaction time in acetonitrile under reflux conditions (entry 4). Replacement of acetonitrile with other solvents such as ethyl acetate, dichloromethane, THF, benzene and dioxane, resulted in lower yields (entries 5–9). Based on these results, the optimal reaction conditions were selected as **1** (1 mmol) and **2** (1.1 mmol) in MeCN (5 mL) under reflux condition for 1.5 h.

**Figure 3:**
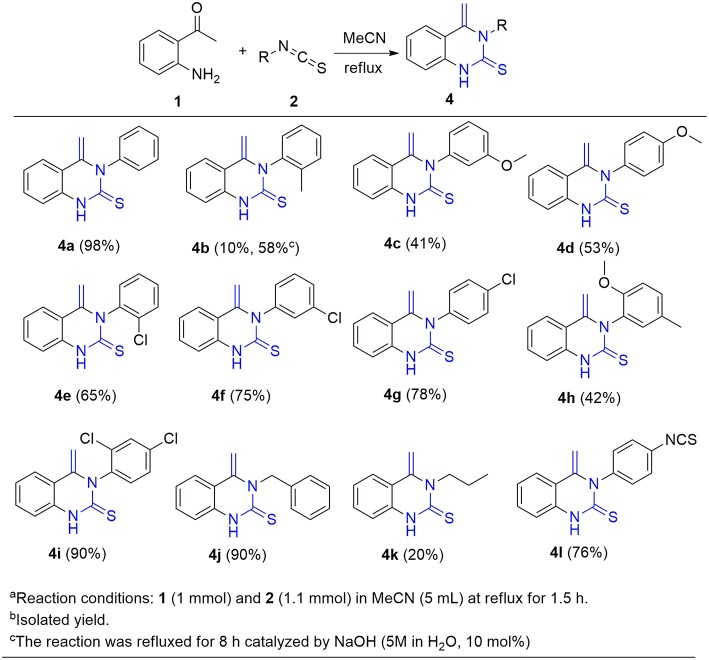
Substrate scope of isothiocyanates^a,b^.

**Scheme 3:**
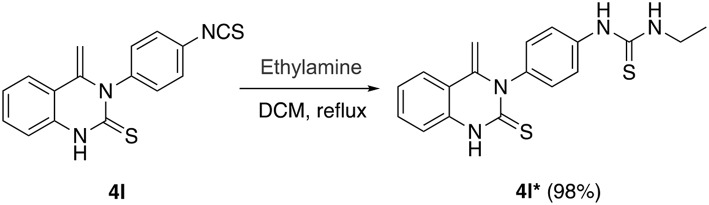
Nucleophilic attack of 4l^*^ by an aliphatic amine.

With these optimized reaction conditions in hand, the substrate scope of this methodology was explored (Figure 3). We found that both electron-donating groups (EDG) and electron-withdrawing groups (EWG) on the aromatic ring were well-tolerated by the reaction conditions. However, the yields of the products attached with EDG were significantly lower than those with EWG (**4b-4d** vs. **4e-4g**). In addition, the stereochemistry also influenced the yield. For example, using *para*- and *meta*-chloro substituted **2** in the reaction provided good yields of **4** whereas *ortho*-chloro substituted **2** gave the corresponding product in only a moderate yield (**4e-g**). The yield sequence followed the order: *para*- > *meta*- > *ortho*-. In similar fashion to mono-substituted analogs, di-substituted isothiocyanates reacted smoothly with 2-aminoacetophenone to furnish the corresponding quinazolinthiones in moderate to high yields (**4h-4i**). Again, the electronic nature of the aryl moiety on the isothiocyanates had a dramatic effect on the product yield. As a result, 2,4-dichloro substituted **2i** gave the corresponding product at 90% yield while the 2-methoxy-5-methyl substituted analog **2h** only offered 42% yield of product **4h**. To further expand the substrate scope, two aliphatic isothiocyanates (**2j**, **2k**) were also investigated for this cycloaddition reaction. Benzyl isothiocyanate (**2j**) was found to be smoothly converted to the corresponding product **4j** in a high yield, whereas propyl isothiocyanate (**2k**) only gave 20% yield of the desired **4k**. In addition, diisothiocyanate (**2l**) was also examined for this conversion, but only one isothiocyanate group was involved in the reaction and a mono-cyclized product **4l** was obtained with the second isothiocyanate not involved in the reaction, even in the presence of an excess of 2-aminoacetophenone **1** (>2 equiv.). This interesting result provides an opportunity for further diversification and amplification with the entire isothiocyanate group. In this regard, compound **4l** was refluxed with ethylamine, a highly nucleophilic amine, to afford compound 4l^*^ with an excellent yield (Scheme 3).

**Scheme 4:**
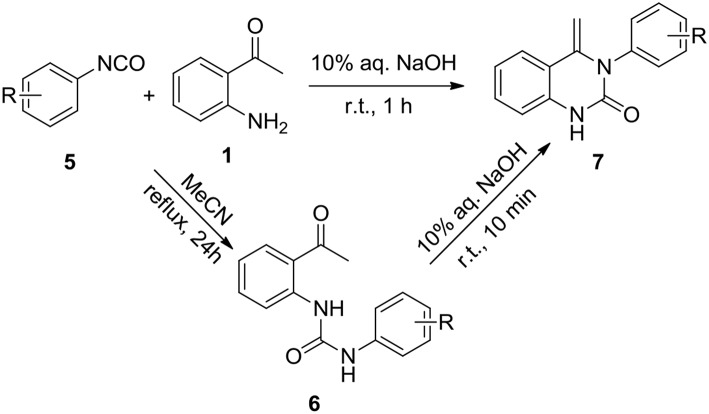
Coupling and annulation reaction of **5** and **6**.

This success led us to further investigate the reaction generality by replacement of isothiocyanates with isocyanates. Under the optimized reaction conditions, the reaction of isocyanate **5** and 2-aminoacetophenone **1** only resulted in the coupling product **6**, even after prolonging the reaction time to 24 h. The annulation compound **7** was not detected (Scheme 4). Pleasingly, compound **6** can undergo an intramolecular cyclization to afford the target 4-methylene-quinazolinone **7** in the presence of NaOH (10 mol%). Encouraged by these results, a subsequent study was undertaken to evaluate a one-pot cascade reaction of isocyanates **5** and 2-aminoacetophenone **1**. In the presence of a catalytic amount of aqueous NaOH (10 mol%), the reaction progressed smoothly to yield the annulation product **7** even at room temperature. Consequently we modified the conditions for the reaction of **1** with **2**, and achieve a significantly improved yield for the production of **4b** (58 vs. 10%, Figure 3).

**Figure 4:**
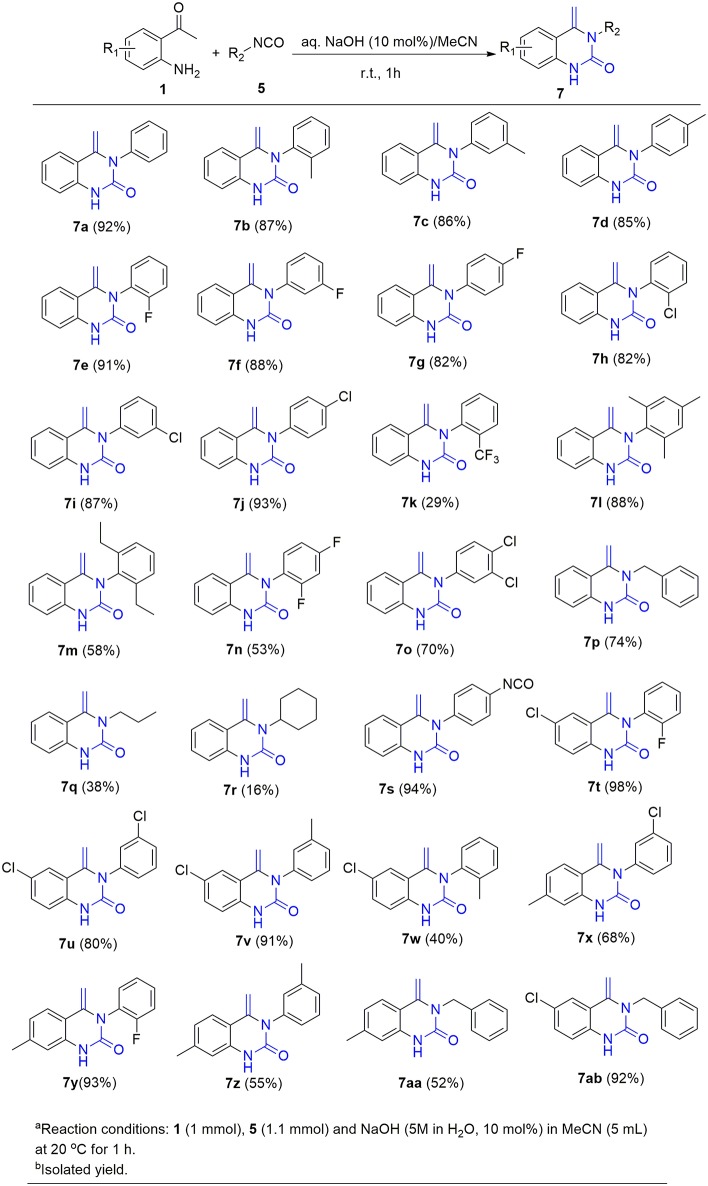
Substrate scope of isocyanates^a,b^.

On the basis of this latter result, the scope of substituted isocyanates was explored (Figure 4). Aromatic isocyanates bearing either an EDG or an EWG group were found to undergo the reaction smoothly and the corresponding 4-methylene-quinazolinones were formed in excellent yields (**7b-7j**). In this case, neither the electronic nature nor position of the functional groups attached to the aromatic ring dramatically influenced the product yield. However, the 2-CF_3_-substituted isocyanate **5k** only gave a moderate yield of the expected product **7k**. Compared to the mono-substituted analogs, di-substituted aryl isocyanates were converted into the corresponding quinolinones in relatively low yields (**7l-7o**). In addition, three aliphatic isocyanates (**5p**-**5r**) were investigated for this annulation reaction. It was found that benzyl isocyanate was converted smoothly to afford the corresponding product **7p** in a high yield, whereas only moderate yields were achieved for **7q** and **7r**. The diisocyanate (**5s**) was also investigated for this conversion. In a similar fashion to the aforementioned reaction with diisothiocyanate (**2l**), only one isocyanate group was involved in the reaction and an excellent yield of mono-cyclized product **7s** was obtained.

The scope of substituted 2-aminoacetophenones **1** was also investigated in this novel cyclization process. 2-Aminoacetophenones bearing either an EWG such as chloro group or an EDG such as methyl group on the aromatic ring, progressed in this reaction smoothly with either aromatic isocyanates or benzyl isocyanate to provide the corresponding quinazolinones in moderate to excellent yield (**7t**-**7ab**).

**Scheme 5:**
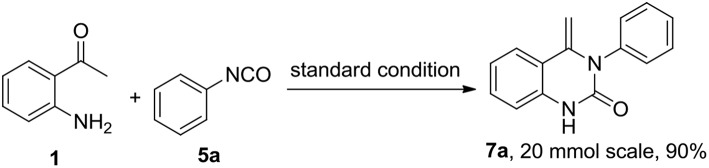
Gram-scale preparation of **7a**.

In order to demonstrate the suitability of this new synthetic methodology for industrial use, an increased scale preparation of **7a** was investigated. Reaction of substrates **1** and **5a** in MeCN (20 mmol, 50 mL) was performed in the presence of NaOH (5 M in H_2_O, 10 mol%) at room temperature. The corresponding product **7a** was afforded in 90% yield, the yield being similar to that of the small-scale reaction (Scheme 5). Moreover, the work-up procedure is simple, as the product precipitated from MeCN on cooling the reaction medium. A pure product was obtained after filtration, without the requirement of further purification.

**Figure 5:**
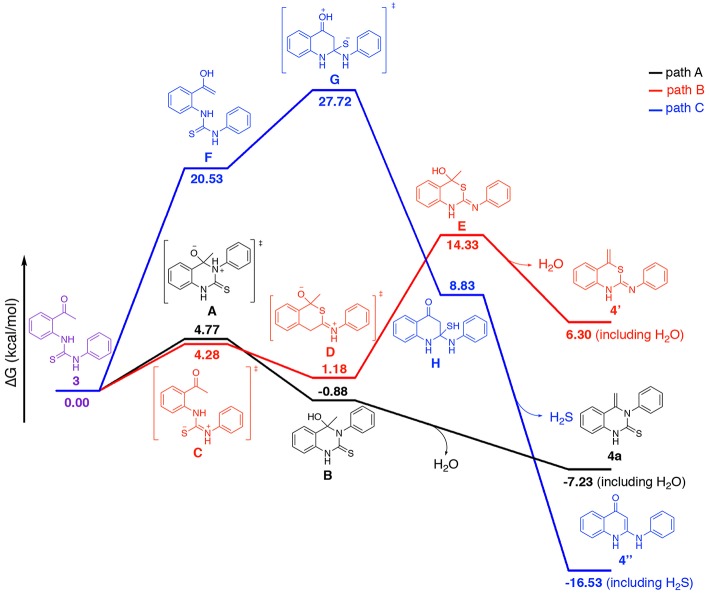
Calculated comparative Gibbs free energy profile for three different mechanism routes. Red, Blue, and Black lines stand for the route of TS and optimized structures Gibbs energy change. Calculated Gibbs free energies relative to **3** at SMD-B3LYP-D3(BJ)/BS1//B3LYP/BS1 [kcal/mol] (see Supporting Information for optimized structures).

As indicated in Scheme 2, the heterocyclization of starting materials **1** and **2**/**5** may in principle lead to at least three different products. To better understand the intramolecular cyclization reaction, we calculated Gibbs free energy of each intermediate and products listed in Scheme 2 by a computational method. Compounds **1** and **2a** were selected as the model substrates for this study. The calculation was based on quantum chemical calculations (SMD-B3LYP-D3(BJ)/BS1//B3LYP/BS1). The geometry of transition states (TSs) and compound **3** in Scheme 2 have been fully optimized in vacuum and the TSs were verified to have only one imaginary frequency vibrational mode that connects the reactants and products. The calculated energy values of the intermediates and products, including dispersion interactions are presented in Figure 5. All stationary points were fully optimized and subjected to frequency analyses. The data demonstrates that paths A and C mechanism routes have a negative value of Gibbs free energy for the final product while the path B mechanism route gives a positive value, thus ruling out the path B mechanism. In the route of path A **3** → **A** → **B** → **4a** (black, Figure 5), there is only one energy barrier at a value of 4.77 kcal/mol. In contrast, there are two high energy barriers (20.53 and 7.19 kcal/mol) in the route of path C **3** → **F** → **G** → **H** → **4″** (blue), rendering the transformation *via* path C difficult. These results indicate that the route of path A is the most probable pathway for this annulation reaction.

## Conclusion

In summary, we have presented a novel preparation of 3-substituted 4-methylene-3,4-dihydroquinazoline-2(1*H*)-thiones/ones from 2-aminoacetophenone and isothiocyanates/isocyanates. The method utilizes commercially available starting materials and is applicable to a large range of compounds. This new synthetic methodology does not require expensive/complicated metal catalysts and the work-up procedure is simple. The novel transformation indicates the feasibility of this pathway in both research and industrial laboratories. Comprehensive quantum chemical calculation studies indicate the probable synthetic route.

## Materials and Methods

### Experimental and Computational Details

All chemicals were obtained from Aladdin (China) as reagent grade and were used as received. Column chromatography purifications were performed on silica gel 60 (0.04–0.063 mm). Melting points were determined using an Electrothermal WRS-1B Digital Melting Point Apparatus and are uncorrected. ^1^H-NMR spectra were recorded using a Bruker (400/500 MHz) NMR spectrometer. ^13^C-NMR spectra were recorded using a Bruker (101/151 MHz) NMR spectrometer. Chemical shifts (δ) are reported in ppm downfield from the internal standard tetramethylsilane (TMS).

Gaussian 09 software (Frisch et al., 2016) packages can implement density functional theory (DFT) conveniently, thus geometry optimizations were carried out with the hybrid B3LYP functional in conjunction with 6-31G++(d,p) basis set for all the atoms of these compounds. Additionally, the geometry optimizations were followed by frequency calculations using the same basis set. Moreover, the effect of dispersion was incorporated using Grimme-D3 approximation during geometry optimizations (Grimme et al., 2010). To further refine the energies, single-point B3LYP calculations including D3 version of Grimme's dispersion with Becke-Johnson damping (D3BJ) (Grimme et al., 2011) were performed with a higher basis set BS1 (BS1 = 6–31++G(d,p) basis set for all atoms). The use of SMD-B3LYP-D3(BJ)/BS1 to calculate compound Gibbs energy was reported in the literature for mechanistic investigation (Markovic et al., 2010; Kleine et al., 2011).

### Chemical Synthesis

#### Synthesis of 4a-4l

A mixture of 2-aminoacetophenone **1** (1 mmol) and isothiocyanatobenzene **2** (1.1 mmol) in acetonitrile (5 ml) was stirred at 82°C for 4 h. After completion, the reaction solvent was reduced to half and upon cooling a white or yellow precipitate was obtained. Recrystallization from ethanol produced white or yellow solid.

##### 4-Methylene-3-phenyl-3,4-dihydroquinazoline-2(1H)-thione (4a)

Yellow solid, m.p. 217~218°C; ^1^H NMR (400 MHz, Chloroform-*d*) δ 9.98 (s, 1H), 7.56 (dd, *J* = 8.3, 6.8 Hz, 2H), 7.52–7.45 (m, 2H), 7.31–7.26 (m, 3H), 7.24 (d, *J* = 1.3 Hz, 1H), 7.07 (td, *J* = 7.7, 1.2 Hz, 1H), 6.91 (dd, *J* = 8.0, 1.1 Hz, 1H), 4.82 (d, *J* = 2.6 Hz, 1H), 3.69 (d, *J* = 2.6 Hz, 1H). ^13^C NMR (101 MHz, Chloroform-*d*) δ 174.18, 141.83, 141.66, 133.23, 130.65, 130.07, 128.94, 128.67, 124.40, 123.83, 118.26, 114.84, 90.14. HRMS (ESI): Calc. for C_15_H_12_N_2_S [M + H]^+^: 253.0794, found 253.2068.

##### 4-Methylene-3-(o-tolyl)-3,4-dihydroquinazoline-2(1H)-thione (4b)

Brown powder, m.p. 214~215°C; ^1^H NMR (500 MHz, DMSO-*d*_6_) δ 11.64 (s, 1H), 7.68 (d, *J* = 8.4 Hz, 1H), 7.40–7.36 (m, 2H), 7.36–7.32 (m, 2H), 7.16–7.11 (m, 2H), 7.09 (dd, *J* = 7.9, 1.2 Hz, 1H), 4.89 (d, *J* = 2.1 Hz, 1H), 3.38 (d, *J* = 2.2 Hz, 1H), 2.13 (s, 3H). ^13^C NMR (126 MHz, DMSO-*d*_6_) δ 173.38, 140.67, 140.62, 135.83, 134.15, 131.68, 131.29, 129.36, 128.86, 128.10, 124.49, 124.42, 117.65, 115.58, 88.04, 17.16. HRMS (ESI) *m/z*: Calc. for C_16_H_14_N_2_S [M + H]^+^: 267.0950, found 257.0933.

##### 3-(3-Methoxyphenyl)-4-methylene-3,4-dihydroquinazoline-2(1H)-thione (4c)

Yellow powder, m.p. 221~225°C; ^1^H NMR (500 MHz, DMSO-*d*_6_) δ 11.63 (s, 1H), 7.66 (dd, *J* = 8.1, 1.3 Hz, 1H), 7.47–7.41 (m, 1H), 7.37 (ddd, *J* = 8.4, 7.3, 1.3 Hz, 1H), 7.14 (dd, *J* = 8.1, 1.2 Hz, 1H), 7.09 (ddd, *J* = 8.3, 7.3, 1.2 Hz, 1H), 7.03–6.97 (m, 1H), 6.83–6.79 (m, 2H), 4.92 (d, *J* = 2.2 Hz, 1H), 3.77 (s, 3H), 3.51 (d, *J* = 2.2 Hz, 1H). ^13^C NMR (126 MHz, DMSO-*d*_6_) δ 173.95, 160.93, 143.12, 141.84, 134.13, 131.20, 130.93, 124.40, 124.36, 121.71, 118.04, 115.51, 115.30, 114.15, 89.25, 55.81. HRMS (ESI) *m/z*: Calc. for C_16_H_14_N_2_OS [M + H]^+^: 283.0900, found 283.0864.

##### 3-(4-Methoxyphenyl)-4-methylene-3,4-dihydroquinazoline-2(1H)-thione (4d)

White powder, m.p. 212~214°C; ^1^H NMR (400 MHz, Chloroform-*d*) δ 9.19 (s, 1H), 7.54 (d, *J* = 8.0 Hz, 1H), 7.33 (t, *J* = 7.9 Hz, 1H), 7.22 (d, *J* = 8.5 Hz, 2H), 7.16–7.04 (m, 3H), 6.87 (s, 1H), 4.85 (d, *J* = 2.1 Hz, 1H), 3.89 (s, 3H), 3.80 (d, *J* = 2.1 Hz, 1H). ^13^C NMR (126 MHz, DMSO-*d*_6_) δ 174.47, 159.01, 142.36, 134.93, 134.14, 131.18, 130.57, 124.44, 124.34, 118.05, 115.50, 115.39, 89.18, 55.76. HRMS (ESI) *m/z*: calc. for C_16_H_14_N_2_OS [M + H]^+^: 283.0900, found 283.0912.

##### 3-(2-Chlorophenyl)-4-methylene-3,4-dihydroquinazoline-2(1H)-thione (4e)

Yellow powder, m.p. 203~205°C; ^1^H NMR (500 MHz, DMSO-*d*_6_) δ 11.76 (s, 1H), 7.70 (d, *J* = 8.0 Hz, 1H), 7.65 (dd, *J* = 7.8, 1.8 Hz, 1H), 7.55–7.45 (m, 2H), 7.44–7.36 (m, 2H), 7.17–7.08 (m, 2H), 4.93 (d, *J* = 2.6 Hz, 1H), 3.38 (d, *J* = 2.6 Hz, 1H). ^13^C NMR (126 MHz, DMSO-*d*_6_) δ 173.56, 140.37, 138.85, 133.99, 132.29, 132.00, 131.38, 130.96, 130.54, 129.24, 124.56, 124.52, 117.66, 115.68, 88.17. HRMS (ESI) *m/z*: calc. for C_15_H_11_ClN_2_S [M + H]^+^: 287.0404, found 287.0389.

##### 3-(3-Chlorophenyl)-4-methylene-3,4-dihydroquinazoline-2(1H)-thione (4f)

White powder, m.p. 234~236°C; ^1^H NMR (400 MHz, DMSO-*d*_6_) δ 11.79 (d, *J* = 2.5 Hz, 1H), 7.69 (t, *J* = 7.7 Hz, 1H), 7.59 (, *J* = 7.9, 2.6 Hz, 1H), 7.56–7.49 (m, 1H), 7.46–7.35 (m, 2H), 7.27 (dt, *J* = 7.8, 1.6 Hz, 1H), 7.21–7.16 (m, 1H), 7.12 (dd, *J* = 9.8, 5.6 Hz, 1H), 4.96 (d, *J* = 4.1, 2.2 Hz, 1H), 3.46 (d, *J* = 2.5 Hz, 1H). ^13^C NMR (101 MHz, DMSO-*d*_6_) δ 173.93, 143.32, 141.91, 134.21, 134.06, 131.86, 131.30, 129.90, 128.81, 128.77, 124.49, 124.42, 118.03, 115.64, 89.35. HRMS (ESI) *m/z*: calc. for C_15_H_11_ClN_2_S [M + H]^+^: 287.0404, found 287.0423.

##### 3-(4-Chlorophenyl)-4-methylene-3,4-dihydroquinazoline-2(1H)-thione (4g)

White powder, m.p. 240~245°C; ^1^H NMR (500 MHz, DMSO-*d*_6_) δ 11.72 (s, 1H), 7.68 (dd, *J* = 8.2, 1.4 Hz, 1H), 7.61–7.57 (m, 2H), 7.38 (ddd, *J* = 8.4, 7.2, 1.3 Hz, 1H), 7.31–7.26 (m, 2H), 7.15 (dd, *J* = 8.1, 1.2 Hz, 1H), 7.10 (ddd, *J* = 8.3, 7.3, 1.3 Hz, 1H), 4.94 (d, *J* = 2.4 Hz, 1H), 3.45 (d, *J* = 2.5 Hz, 1H). ^13^C NMR (126 MHz, DMSO-*d*_6_) δ 174.01, 141.96, 141.00, 134.06, 133.08, 131.74, 131.30, 130.43, 124.49, 124.45, 118.00, 115.61, 89.33. HRMS (ESI) *m/z*: calc. for C_15_H_11_ClN_2_S [M + H]^+^: 287.0404, found 287.0367.

##### 3-(2-Methoxy-6-methylphenyl)-4-methylene-3,4-dihydroquinazoline-2(1H)-thione (4h)

Yellow powder, m.p. 236~237°C; ^1^H NMR (500 MHz, DMSO-*d*_6_) δ 11.54 (s, 1H), 7.64 (dd, *J* = 8.1, 1.3 Hz, 1H), 7.35 (ddd, *J* = 8.4, 7.3, 1.3 Hz, 1H), 7.23–7.19 (m, 1H), 7.12 (dd, *J* = 8.2, 1.2 Hz, 1H), 7.10–7.05 (m, 2H), 6.95 (dd, *J* = 2.2, 0.8 Hz, 1H), 4.82 (d, *J* = 2.1 Hz, 1H), 3.71 (s, 3H), 3.50 (d, *J* = 2.1 Hz, 1H), 2.28 (s, 3H). ^13^C NMR (126 MHz, DMSO-*d*_6_) δ 174.12, 152.82, 140.82, 134.17, 131.13, 130.83, 130.60, 130.52, 129.80, 124.42, 124.30, 117.86, 115.47, 113.43, 87.98, 56.21, 20.42. HRMS (ESI) *m/z*: calc. for C_17_H_16_N_2_OS [M + H]^+^: 297.1056, found 297.1023.

##### 3-(2,4-Dichlorophenyl)-4-methylene-3,4-dihydroquinazoline-2(1H)-thione (4i)

White powder, m.p. 236~237°C; ^1^H NMR (500 MHz, DMSO-*d*_6_) δ 11.82 (s, 1H), 7.85 (d, *J* = 2.3 Hz, 1H), 7.71 (dd, *J* = 8.1, 1.3 Hz, 1H), 7.60 (dd, *J* = 8.5, 2.4 Hz, 1H), 7.48 (d, *J* = 8.5 Hz, 1H), 7.39 (ddd, *J* = 8.4, 7.2, 1.3 Hz, 1H), 7.18–7.13 (m, 1H), 7.11 (dd, *J* = 8.1, 1.1 Hz, 1H), 4.96 (d, *J* = 2.7 Hz, 1H), 3.45 (d, *J* = 2.8 Hz, 1H). ^13^C NMR (126 MHz, DMSO-*d*_6_) δ 173.48, 140.17, 138.00, 134.16, 133.91, 133.65, 133.46, 131.44, 130.64, 129.54, 124.66, 124.55, 117.61, 115.75, 88.33. HRMS (ESI) *m/z*: calc. for C_15_H_10_Cl_2_N_2_S [M + H]^+^: 321.0015, found 321.0023.

##### 3-Benzyl-4-methylene-3,4-dihydroquinazoline-2(1H)-thione (4j)

White powder, m.p. 241~242°C (Morgenstern and Richter, 1992, 215~217°C); ^1^H NMR (500 MHz, DMSO-*d*_6_) δ 11.65 (s, 1H), 7.60 (dd, *J* = 8.1, 1.3 Hz, 1H), 7.37–7.27 (m, 5H), 7.27–7.21 (m, 1H), 7.14 (dd, *J* = 8.1, 1.2 Hz, 1H), 7.07 (ddd, *J* = 8.3, 7.3, 1.2 Hz, 1H), 5.68 (s, 2H), 4.98 (d, *J* = 2.9 Hz, 1H), 4.31 (d, *J* = 2.9 Hz, 1H). ^13^C NMR (126 MHz, DMSO-*d*_6_) δ 174.87, 138.11, 136.39, 133.73, 131.01, 128.93, 127.28, 126.59, 124.59, 124.27, 118.02, 115.42, 89.39, 52.81. HRMS (ESI) *m/z*: calc. for C_16_H_14_N_2_S [M + H]^+^: 267.0950, found 267.0932.

##### 4-Methylene-3-propyl-3,4-dihydroquinazoline-2(1H)-thione (4k)

White powder, m.p. 168~169°C; ^1^H NMR (500 MHz, DMSO-*d*_6_) δ 11.35 (s, 1H), 7.65 (dd, *J* = 8.3, 1.3 Hz, 1H), 7.33–7.28 (m, 1H), 7.08–7.04 (m, 2H), 5.05 (d, *J* = 2.9 Hz, 1H), 4.52 (d, *J* = 2.9 Hz, 1H), 4.25 (s, 2H), 1.78–1.68 (m, 2H), 0.92 (t, *J* = 7.4 Hz, 3H). ^13^C NMR (126 MHz, DMSO-*d*_6_) δ 173.70, 138.33, 133.85, 130.88, 124.37, 124.34, 118.06, 115.18, 87.82, 50.78, 18.16, 11.31. HRMS (ESI) *m/z*: (M+H^+^); calc. for C_12_H_14_N_2_S [M + H]^+^: 219.0950, found 219.0938.

##### 3-(4-Isothiocyanatophenyl)-4-methylene-3,4-dihydroquinazoline-2(1H)-thione (4l)

Yellow powder, m.p. 236~239°C; ^1^H NMR (500 MHz, DMSO-*d*_6_) δ 11.74 (s, 1H), 7.68 (dd, *J* = 8.1, 1.3 Hz, 1H), 7.61–7.56 (m, 2H), 7.38 (ddd, *J* = 9.5, 6.7, 1.4 Hz, 1H), 7.35–7.32 (m, 2H), 7.15 (dd, *J* = 8.1, 1.2 Hz, 1H), 7.10 (ddd, *J* = 8.3, 7.2, 1.2 Hz, 1H), 4.94 (d, *J* = 2.4 Hz, 1H), 3.43 (d, *J* = 2.5 Hz, 1H). ^13^C NMR (126 MHz, DMSO-*d*_6_) δ 173.92, 141.93, 141.31, 134.70, 134.05, 131.51, 131.32, 130.13, 128.00, 124.50, 124.45, 118.00, 115.63, 89.35. HRMS (ESI) *m/z*: calc. for C_16_H_12_N_3_S_2_ [M + H]^+^: 310.0467, found 310.0421.

#### Synthesis of 4l^*^

A mixture of **4l** (1 mmol) and ethylamine (1.1 mmol) in dichloromethane (5 ml) were refluxed for 4 h. After cooling it down to the room temperature, the precipitate was filtered and washed with ethyl acetate. Recrystallization from ethanol produced yellow solid.

##### 1-Ethyl-3-(4-(4-methylene-2-thioxo-1,4-dihydroquinazolin-3(2H)-yl)phenyl)thiourea (4l^*^)

Yellow solid. m.p. 197~202°C; 1H NMR (400 MHz, DMSO-d_6_) δ 10.33 (s, 1H), 9.69 (s, 1H), 7.94 (s, 1H), 7.67 (d, J = 8.0 Hz, 1H), 7.61 (d, J = 8.4 Hz, 2H), 7.42–7.33 (m, 1H), 7.16–7.07 (m, 3H), 4.93 (d, J = 2.2 Hz, 1H), 3.55 (d, J = 2.1 Hz, 1H), 3.50 (q, J = 6.6 Hz, 2H), 1.15 (t, J = 7.2 Hz, 3H). 13C NMR (101 MHz, DMSO-d6) δ 180.40, 174.19, 142.15, 139.56, 137.43, 134.11, 131.21, 129.62, 124.45, 124.38, 121.66, 118.04, 115.51, 89.45, 39.09, 14.61. HRMS (ESI) m/z: calc. for C_18_H_18_N_4_S_2_ [M + H]^+^: 355.1046, found 355.1057.

#### Synthesis of 6a and 6r

A mixture of 2-aminoacetophenone (1 mmol) and isocyanatobenzene/isocyanatocyclohexane (1.1 mmol) in acetonitrile (5 ml) was stirred at 82°C for 4 h. After completion, the reaction solvent was reduced to half and upon cooling a white or yellow precipitate was obtained. Recrystallization from ethanol produced white solid.

##### 1-(2-Acetylphenyl)-3-phenylurea (6a)

White solid. m.p. 218–221°C (Iwamura et al., 1980, 223°C); ^1^H NMR (400 MHz, Chloroform-*d*) δ 11.34 (s, 1H), 8.60 (dd, *J* = 8.6, 1.2 Hz, 1H), 7.85 (dd, *J* = 8.1, 1.6 Hz, 1H), 7.52 (ddd, *J* = 8.6, 7.1, 1.6 Hz, 1H), 7.48–7.39 (m, 2H), 7.38–7.29 (m, 2H), 7.16–7.08 (m, 1H), 7.03 (ddd, *J* = 8.3, 7.2, 1.2 Hz, 1H), 6.94 (s, 1H), 2.63 (s, 3H). ^13^C NMR (101 MHz, Chloroform-*d*) δ 203.04, 152.81, 142.43, 137.99, 135.18, 131.68, 129.16, 124.09, 121.23, 120.99, 120.84, 120.19, 28.49. HRMS (ESI) m/z: calc. for C_15_H_13_N_2_O_2_ [M + H]^+^:255.1128, found 255.1099.

##### 1-(2-Acetylphenyl)-3-cyclohexylurea (6r)

White solid. m.p. 175–178°C; ^1^H NMR (500 MHz, DMSO-*d*_6_) δ 10.43 (s, 1H), 8.38 (dd, *J* = 8.6, 1.2 Hz, 1H), 7.94 (dd, *J* = 8.0, 1.6 Hz, 1H), 7.48 (ddd, *J* = 8.7, 7.1, 1.6 Hz, 1H), 7.36 (br s, 1H), 6.99 (ddd, *J* = 8.2, 7.2, 1.2 Hz, 1H), 3.48–3.38 (m, 1H), 2.61 (s, 3H), 1.84–1.75 (m, 2H), 1.74–1.65 (m, 2H), 1.34–1.22 (m, 2H), 1.22–1.00 (m, 4H). ^13^C NMR (126 MHz, DMSO-*d*_6_) δ 202.59, 154.47, 142.67, 134.55, 132.40, 121.79, 120.34, 119.91, 48.74, 33.32, 29.16, 25.75, 25.15. HRMS (ESI) m/z: calc. for C_15_H_20_N_2_O_2_ [M + H]^+^: 261.1598, found 261.1572.

#### Synthesis of 7a-7ab

A mixture of 2-aminoacetophenone **1** (1 mmol), isocyanatobenzene **5** (1.1 mmol) and a drop of aqueous NaOH solution (5 M, 10 mol%) in acetonitrile (5 ml) were stirred at room temperature. After completion, the reaction solvent was reduced to half and upon cooling a white or yellow precipitate was obtained. Recrystallization from ethanol produced white solid.

##### 4-Methylene-3-phenyl-3,4-dihydroquinazolin-2(1H)-one (7a)

White solid. m.p. 186~188°C (Sbei et al., 2018, 188~190°C); ^1^H NMR (400 MHz, Chloroform-*d*) δ 8.18 (s, 1H), 7.59–7.49 (m, 3H), 7.47–7.40 (m, 1H), 7.35–7.28 (m, 2H), 7.24 (dd, *J* = 7.9, 1.3 Hz, 1H), 7.06–6.96 (m, 1H), 6.72 (dd, *J* = 8.1, 1.2 Hz, 1H), 4.74 (d, *J* = 2.2 Hz, 1H), 3.68 (d, *J* = 2.2 Hz, 1H). ^13^C NMR (101 MHz, Chloroform-*d*) δ 150.88, 143.31, 138.42, 135.16, 130.27, 129.94, 129.19, 128.34, 123.94, 122.73, 117.03, 115.09, 87.43. HRMS (ESI) *m/z*: calc. for C_15_H_12_N_2_O [M + H]^+^: 237.1022, found 237.1908.

##### 4-Methylene-3-(o-tolyl)-3,4-dihydroquinazolin-2(1H)-one (7b)

White solid. m.p. 204~207°C; ^1^H NMR (500 MHz, DMSO-*d*_6_) δ 10.30 (s, 1H), 7.68 (dd, *J* = 8.1, 1.3 Hz, 1H), 7.42–7.37 (m, 1H), 7.37–7.30 (m, 3H), 7.19–7.15 (m, 1H), 7.01 (td, *J* = 7.8, 1.2 Hz, 1H), 6.96 (dd, *J* = 8.1, 1.2 Hz, 1H), 4.75 (d, *J* = 1.8 Hz, 1H), 3.33 (d, *J* = 1.8 Hz, 1H), 2.11 (s, 3H). ^13^C NMR (126 MHz, DMSO-*d*_6_) δ 149.27, 142.44, 137.75, 136.56, 136.42, 131.50, 130.90, 129.77, 128.77, 127.89, 124.63, 122.61, 116.29, 115.29, 85.21, 17.19. HRMS (ESI) *m/z*: calc. for C_16_H_14_N_2_O [M + H]^+^: 251.1179, found 251.1121.

##### 4-Methylene-3-(m-tolyl)-3,4-dihydroquinazolin-2(1H)-one (7c)

Yellow powder. m.p. 206~208°C; ^1^H NMR (500 MHz, DMSO-*d*_6_) δ 10.27 (s, 1H), 7.65 (dd, *J* = 8.0, 1.3 Hz, 1H), 7.40 (t, *J* = 7.7 Hz, 1H), 7.31 (ddd, *J* = 8.4, 6.0, 1.4 Hz, 1H), 7.25–7.22 (m, 1H), 7.09–7.03 (m, 2H), 6.99 (ddd, *J* = 8.3, 7.2, 1.2 Hz, 1H), 6.95 (dd, *J* = 8.1, 1.2 Hz, 1H), 4.76 (d, *J* = 1.8 Hz, 1H), 3.43 (d, *J* = 1.8 Hz, 1H), 2.35 (s, 3H). ^13^C NMR (126 MHz, DMSO-*d*_6_) δ 149.71, 143.72, 139.63, 139.04, 136.37, 130.82, 130.16, 129.92, 129.07, 126.71, 124.53, 122.57, 116.59, 115.22, 86.38, 21.24. HRMS (ESI) *m/z*: calc. for C_16_H_14_N_2_O [M + H]^+^: 251.1179, found 251.1098.

##### 4-Methylene-3-(p-tolyl)-3,4-dihydroquinazolin-2(1H)-one (7d)

Yellow powder. m.p. 222~224°C; ^1^H NMR (500 MHz, DMSO-*d*_6_) δ 10.26 (s, 1H), 7.66 (dd, *J* = 8.1, 1.3 Hz, 1H), 7.36 (d, *J* = 8.4 Hz, 1H), 7.31 (td, *J* = 8.0 Hz, 1.2 Hz, 1H), 7.15–7.11 (m, 2H), 7.05 (d, *J* = 8.3 Hz, 1H), 7.00 (td, *J* = 8.1 Hz, 1.2 Hz, 1H), 6.94 (d, *J* = 7.9 Hz, 1H), 4.76 (d, *J* = 1.8 Hz, 1H), 3.43 (d, *J* = 1.8 Hz, 1H), 2.37 (s, 3H). ^13^C NMR (126 MHz, DMSO-*d*_6_) δ 149.77, 143.81, 138.18, 137.74, 136.49, 136.36, 130.83, 130.65, 129.49, 124.56, 122.56, 118.61, 116.58, 115.20, 86.25, 21.21. HRMS (ESI) *m/z*: calc. for C_16_H_14_N_2_O [M + H]^+^: 251.1179, found 251.1093.

##### 3-(2-Fluorophenyl)-4-methylene-3,4-dihydroquinazolin-2(1H)-one (7e)

Light yellow powder. m.p. 220~224°C; ^1^H NMR (500 MHz, DMSO-*d*_6_) δ 10.42 (s, 1H), 7.69 (dd, *J* = 8.1, 1.3 Hz, 1H), 7.55–7.48 (m, 1H), 7.46–7.39 (m, 2H), 7.39–7.31 (m, 2H), 7.03 (ddd, *J* = 8.3, 7.3, 1.2 Hz, 1H), 6.97 (dd, *J* = 8.1, 1.3 Hz, 1H), 4.82 (d, *J* = 2.2 Hz, 1H), 3.48 (dd, *J* = 2.2, 0.8 Hz, 1H). ^13^C NMR (126 MHz, DMSO-*d*_6_) δ158.65 (d, *J* = 248 Hz), 149.25, 142.67, 136.14, 132.07, 131.07, 130.98 (d, *J* = 7.6 Hz), 126.12 (d, *J* = 12.6 Hz), 125.97 (d, *J* = 3.8 Hz), 124.66, 122.85, 117.15 (d, *J* = 18.9 Hz), 116.21, 115.41, 85.80. HRMS (ESI) *m/z*: calc. for C_15_H_11_FN_2_O [M + H]^+^: 255.0928, found 255.0876.

##### 3-(3-Fluorophenyl)-4-methylene-3,4-dihydroquinazolin-2(1H)-one (7f)

Brown powder. m.p. 228~230°C; ^1^H NMR (600 MHz, DMSO-*d*_6_) δ 10.37 (s, 1H), 7.68 (dd, *J* = 8.1, 1.3 Hz, 1H), 7.60–7.54 (m, 1H), 7.35–7.27 (m, 2H), 7.24 (dt, *J* = 9.7, 2.2 Hz, 1H), 7.15 (dd, *J* = 7.8, 1.0 Hz, 1H), 7.01 (ddd, *J* = 8.3, 7.4, 1.2 Hz, 1H), 6.95 (dd, *J* = 8.1, 1.2 Hz, 1H), 4.81 (d, *J* = 2.1 Hz, 1H), 3.44 (d, *J* = 2.1 Hz, 1H). ^13^C NMR (151 MHz, DMSO-*d*_6_) δ 163.18 (d, J = 245 Hz), 149.52, 143.48, 140.70 (d, *J* = 9.1 Hz), 136.23, 131.64 (d, *J* = 9.1 Hz), 130.93, 126.23 (d, *J* = 3.0 Hz), 124.57, 122.67, 117.32 (d, *J* = 22.7 Hz), 116.51, 115.56 (d, *J* = 21 Hz), 115.28, 86.47. HRMS (ESI) *m/z*: calc. for C_15_H_11_FN_2_O [M + H]^+^: 255.0928, found 255.0885.

##### 3-(4-Fluorophenyl)-4-methylene-3,4-dihydroquinazolin-2(1H)-one (7g)

Light yellow powder. m.p. 245~247°C (Molina et al., 1993, 136~138°C); ^1^H NMR (500 MHz, DMSO-*d*_6_) δ 10.33 (s, 1H), 7.67 (dd, *J* = 8.1, 1.3 Hz, 1H), 7.36–7.30 (m, 5H), 7.01 (ddd, *J* = 8.2, 7.3, 1.2 Hz, 1H), 6.95 (dd, *J* = 8.1, 1.2 Hz, 1H), 4.79 (d, *J* = 2.0 Hz, 1H), 3.42 (d, *J* = 2.0 Hz, 1H). ^13^C NMR (126 MHz, DMSO-*d*_6_) δ 161.80 (d, *J* = 246 Hz), 149.74, 143.82, 136.29, 135.31 (d, *J* = 2.5 Hz), 131.97 (d, *J* = 8.8 Hz), 130.90, 124.58, 122.63, 117.02 (d, *J* = 22.7 Hz), 116.54, 115.27, 86.38. HRMS (ESI) *m/z*: calc. for C_15_H_11_FN_2_O [M + H]^+^: 255.0928, found 255.0943.

##### 3-(2-Chlorophenyl)-4-methylene-3,4-dihydroquinazolin-2(1H)-one (7h)

Light yellow powder. m.p. 229~231°C; ^1^H NMR (400 MHz, DMSO-*d*_6_) δ 10.53 (s, 1H), 7.71 (ddd, *J* = 9.3, 7.2, 1.9 Hz, 2H), 7.58–7.44 (m, 3H), 7.36 (ddd, *J* = 8.3, 7.4, 1.3 Hz, 1H), 7.09–6.98 (m, 2H), 4.82 (d, *J* = 2.2 Hz, 1H), 3.35 (d, *J* = 2.2 Hz, 1H). ^13^C NMR (101 MHz, DMSO-*d*_6_) δ 149.10, 142.19, 136.30, 136.22, 133.08, 132.16, 131.00, 130.84, 130.55, 129.17, 124.64, 122.75, 116.26, 115.47, 85.50. HRMS (ESI) *m/z*: calc. for C_15_H_11_ClN_2_O [M + H]^+^: 271.0633, found 271.0598.

##### 3-(3-Chlorophenyl)-4-methylene-3,4-dihydroquinazolin-2(1H)-one (7i)

White powder. m.p. 227~229°C; ^1^H NMR (400 MHz, DMSO-*d*_6_) δ 10.46 (s, 1H), 7.70 (dd, *J* = 8.1, 1.3 Hz, 1H), 7.63–7.51 (m, 2H), 7.46 (t, *J* = 2.0 Hz, 1H), 7.39–7.29 (m, 2H), 7.08–6.95 (m, 2H), 4.84 (d, *J* = 2.1 Hz, 1H), 3.44 (d, *J* = 2.1 Hz, 1H). ^13^C NMR (101 MHz, DMSO-*d*_6_) δ 149.57, 143.58, 140.56, 136.28, 134.12, 131.75, 130.95, 130.12, 128.91, 128.67, 124.58, 122.69, 116.54, 115.36, 86.54. HRMS (ESI) *m/z*: calc. for C_15_H_11_ClN_2_O [M + H]^+^: 271.0633, found 271.0569.

##### 3-(4-Chlorophenyl)-4-methylene-3,4-dihydroquinazolin-2(1H)-one (7j)

Light green powder. m.p. 223~225°C (Sbei et al., 2018, 214~217°C); ^1^H NMR (400 MHz, DMSO-*d*_6_) δ 10.42 (s, 1H), 7.70 (dd, *J* = 8.1, 1.3 Hz, 1H), 7.64–7.57 (m, 2H), 7.40–7.31 (m, 3H), 7.08–6.96 (m, 2H), 4.83 (d, *J* = 2.1 Hz, 1H), 3.45 (d, *J* = 2.1 Hz, 1H). ^13^C NMR (101 MHz, DMSO-*d*_6_) δ 149.61, 143.63, 138.05, 136.29, 133.00, 131.90, 130.93, 130.27, 124.58, 122.67, 116.54, 115.33, 86.49. HRMS (ESI) *m/z*: calc. for C_15_H_11_ClN_2_O [M + H]^+^: 271.0633, found 271.0896.

##### 4-Methylene-3-(2-(trifluoromethyl)phenyl)-3,4-dihydroquinazolin-2(1H)-one (7k)

Yellow solid. m.p. 309~312°C; ^1^H NMR (500 MHz, DMSO-*d*_6_) δ 10.42 (s, 1H), 7.91 (dd, *J* = 7.9, 1.5 Hz, 1H), 7.86 (td, *J* = 7.8, 1.5 Hz, 1H), 7.70 (td, *J* = 8.5, 1.4 Hz, 2H), 7.53 (d, *J* = 7.8 Hz, 1H), 7.34 (ddd, *J* = 7.8, 7.3, 1.3 Hz, 1H), 7.02 (ddd, *J* = 7.8, 7.4, 1.3 Hz, 1H), 6.97 (dd, *J* = 8.1, 1.3 Hz, 1H), 4.84 (d, *J* = 2.3 Hz, 1H), 3.26 (d, *J* = 2.4 Hz, 1H). ^13^C NMR (126 MHz, DMSO-*d*_6_) δ 149.53, 143.52, 136.70, 136.12, 134.73, 133.11, 130.98, 129.79, 128.16 (m), 127.83, 124.87, 124.53, 122.76, 116.34, 115.40, 86.66. HRMS (ESI) *m/z*: calc. for C_16_H_12_F_3_N_2_O [M + H]^+^: 305.0896, found 305.0822.

##### 3-Mesityl-4-methylene-3,4-dihydroquinazolin-2(1H)-one (7l)

White powder. m.p. 230~232°C; ^1^H NMR (500 MHz, DMSO-*d*_6_) δ 10.31 (s, 1H), 7.69 (dd, *J* = 8.1, 1.3 Hz, 1H), 7.32 (ddd, *J* = 8.3, 7.3, 1.3 Hz, 1H), 7.00 (d, *J* = 4.7 Hz, 3H), 6.99–6.95 (m, 1H), 4.70 (d, *J* = 1.7 Hz, 1H), 3.37 (d, *J* = 1.6 Hz, 1H), 2.29 (s, 3H), 2.04 (s, 6H). ^13^C NMR (126 MHz, DMSO-*d*_6_) δ 148.95, 141.08, 137.57, 136.51, 135.94, 133.93, 130.90, 129.66, 124.71, 122.59, 116.03, 115.32, 83.80, 21.07, 17.37. HRMS (ESI) *m/z*: calc. for C_18_H_18_N_2_O [M + H]^+^: 279.1492, found 279.1421.

##### 3-(2,6-Diethylphenyl)-4-methylene-3,4-dihydroquinazolin-2(1H)-one (7m)

White solid. m.p. 166~168°C; ^1^H NMR (500 MHz, DMSO-*d*_6_) δ 10.33 (s, 1H), 7.69 (dd, *J* = 8.2, 1.3 Hz, 1H), 7.39–7.29 (m, 2H), 7.25 (d, *J* = 7.6 Hz, 2H), 7.01 (ddd, *J* = 8.3, 7.3, 1.2 Hz, 1H), 6.97 (dd, *J* = 8.1, 1.2 Hz, 1H), 4.74 (d, *J* = 1.7 Hz, 1H), 3.31 (d, *J* = 1.8 Hz, 1H), 2.42 (q, *J* = 7.5 Hz, 4H), 1.12 (t, *J* = 7.6 Hz, 6H). ^13^C NMR (126 MHz, DMSO-*d*_6_) δ 149.43, 142.12, 141.77, 136.44, 135.36, 132.56, 130.97, 128.88, 127.15, 124.68, 122.66, 120.86, 116.00, 115.32, 84.83, 24.84, 23.79, 15.07, 14.50. HRMS (ESI) *m/z*: calc. for C_19_H_20_N_2_O [M + H]^+^: 293.1648, found 293.1606.

##### 3-(2,4-Difluorophenyl)-4-methylene-3,4-dihydroquinazolin-2(1H)-one (7n)

White powder. m.p. 226~228°C; ^1^H NMR (500 MHz, DMSO-*d*_6_) δ 10.46 (s, 1H), 7.70 (dd, *J* = 8.0, 1.3 Hz, 1H), 7.55–7.47 (m, 2H), 7.34 (ddd, *J* = 8.4, 7.3, 1.3 Hz, 1H), 7.28–7.22 (m, 1H), 7.03 (ddd, *J* = 8.2, 7.3, 1.2 Hz, 1H), 6.97 (dd, *J* = 8.1, 1.2 Hz, 1H), 4.84 (d, *J* = 2.4 Hz, 1H), 3.52 (d, *J* = 2.3 Hz, 1H). ^13^C NMR (126 MHz, DMSO-*d*_6_) δ 162.30 (dd, *J* = 247.5, 11.8 Hz), 158.86 (dd, *J* = 251.1, 13.2 Hz), 149.26, 142.64, 136.08, 133.28 (dd, *J* = 9.9, 2.1 Hz), 131.11, 124.69, 122.89, 122.70 (dd, *J* = 13.4, 4.0 Hz), 116.18, 115.44, 113.07 (dd, *J* = 22.5, 3.6 Hz), 105.78 (dd, *J* = 27.1, 24.1 Hz), 85.90. HRMS (ESI) *m/z*: (M+H^+^); calc. for C_15_H_10_F_2_N_2_O [M + H]^+^: 273.0834, found 273.0789.

##### 3-(3,4-Dichlorophenyl)-4-methylene-3,4-dihydroquinazolin-2(1H)-one (7o)

White powder. m.p. 234~236°C; ^1^H NMR (400 MHz, DMSO-*d*_6_) δ 10.45 (s, 1H), 7.82 (dd, *J* = 8.5, 1.2 Hz, 1H), 7.76–7.67 (m, 2H), 7.41–7.31 (m, 2H), 7.09–7.00 (m, 1H), 6.98 (dd, *J* = 8.1, 1.2 Hz, 1H), 4.85 (d, *J* = 2.3 Hz, 1H), 3.49 (d, *J* = 2.3 Hz, 1H). ^13^C NMR (101 MHz, DMSO-*d*_6_) δ 149.53, 143.45, 139.15, 136.28, 132.36, 132.08, 131.33, 130.97, 130.67, 124.59, 122.71, 116.53, 115.41, 86.71. HRMS (ESI) *m/z*: calc. for C_15_H_10_Cl_2_N_2_O [M + H]^+^: 305.0243, found 305.0185.

##### 3-Benzyl-4-methylene-3,4-dihydroquinazolin-2(1H)-one (7p)

White solid. m.p. 212~214°C; ^1^H NMR (500 MHz, DMSO-*d*_6_) δ 10.28 (s, 1H), 7.62 (d, *J* = 8.1 Hz, 1H), 7.36–7.19 (m, 6H), 7.00–6.92 (m, 2H), 5.01 (s, 2H), 4.82 (d, *J* = 2.4 Hz, 1H), 4.12 (d, *J* = 2.5 Hz, 1H). ^13^C NMR (126 MHz, DMSO-*d*_6_) δ 150.60, 140.23, 137.50, 136.08, 130.65, 128.92, 127.21, 126.81, 124.40, 122.62, 116.46, 115.12, 85.70, 46.30. HRMS (ESI) *m/z*: calc. for C_16_H_14_N_2_O [M + H]^+^: 251.1179, found 251.1085.

##### 4-Methylene-3-propyl-3,4-dihydroquinazolin-2(1H)-one (7q)

Brown powder. m.p. 114~117°C; ^1^H NMR (500 MHz, DMSO-*d*_6_) δ 10.06 (s, 1H), 7.65 (dd, *J* = 8.2, 1.3 Hz, 1H), 7.26 (ddd, *J* = 8.4, 7.2, 1.3 Hz, 1H), 6.96 (ddd, *J* = 8.2, 7.3, 1.3 Hz, 1H), 6.87 (dd, *J* = 8.1, 1.2 Hz, 1H), 4.87 (d, *J* = 2.3 Hz, 1H), 4.28 (d, *J* = 2.4 Hz, 1H), 3.70 (t, *J* = 7.6 Hz, 2H), 1.65–1.55 (m, 2H), 0.89 (t, *J* = 7.4 Hz, 3H). ^13^C NMR (126 MHz, DMSO-*d*_6_) δ 150.07, 140.25, 136.15, 130.51, 124.45, 122.41, 116.51, 114.90, 84.02, 44.26, 18.91, 11.57. HRMS (ESI) *m/z*: calc. for C_12_H_14_N_2_S [M + H]^+^: 203.1179, found 203.1089.

##### 3-Cyclohexyl-4-methylene-3,4-dihydroquinazolin-2(1H)-one (7r)

White powder. m.p. 138~140°C; ^1^H NMR (400 MHz, DMSO-*d*_6_) δ 9.90 (s, 1H), 7.58 (dd, *J* = 8.0, 1.3 Hz, 1H), 7.25 (td, *J* = 7.6, 1.3 Hz, 1H), 7.01–6.89 (m, 1H), 6.87 (dd, *J* = 8.1, 1.2 Hz, 1H), 4.89 (d, *J* = 2.4 Hz, 1H), 4.53 (d, *J* = 2.4 Hz, 1H), 3.84 (tt, *J* = 12.0, 3.6 Hz, 1H), 2.39 (qd, *J* = 12.5, 3.6 Hz, 2H), 1.82–1.62 (m, 4H), 1.45–1.01 (m, 4H). ^13^C NMR (101 MHz, DMSO-*d*_6_) δ 150.56, 142.08, 136.27, 130.15, 124.40, 122.27, 118.46, 114.34, 88.15, 58.37, 29.02, 26.42, 25.70. HRMS (ESI) *m/z*: calc. for C_15_H_18_N_2_O [M + H]^+^: 243.1492, found 243.1464.

##### 3-(4-Isocyanatophenyl)-4-methylene-3,4-dihydroquinazolin-2(1H)-one (7s)

Light yellow powder. m.p. 230~232°C; ^1^H NMR (500 MHz, DMSO-*d*_6_) δ 11.75 (s, 1H), 7.69 (dd, *J* = 8.1, 1.4 Hz, 1H), 7.57 (t, *J* = 7.9 Hz, 1H), 7.53–7.49 (m, 1H), 7.43–7.34 (m, 2H), 7.28–7.22 (m, 1H), 7.15 (dd, *J* = 8.1, 1.3 Hz, 1H), 7.13–7.08 (m, 1H), 4.95 (d, *J* = 2.5 Hz, 1H), 3.44 (d, *J* = 2.5 Hz, 1H). ^13^C NMR (126 MHz, DMSO-*d*_6_) δ 173.93, 143.32, 141.91, 134.18, 134.05, 131.85, 131.30, 129.90, 128.81, 128.79, 124.49, 124.43, 118.03, 115.63, 89.34. HRMS (ESI) *m/z*: calc. for C_16_H_11_N_3_O_2_ [M + H]^+^: 278.0924, found 278.0882.

##### 6-Chloro-3-(2-fluorophenyl)-4-methylene-3,4-dihydroquinazolin-2(1H)-one (7t)

White powder. m.p. 258.9~261.3°C; ^1^H NMR (400 MHz, DMSO-*d*_6_) δ 10.69 (s, 1H), 7.80 (d, *J* = 2.3 Hz, 1H), 7.56–7.48 (m, 1H), 7.47–7.32 (m, 4H), 6.99 (dd, *J* = 8.7, 1.9 Hz, 1H), 4.94 (d, *J* = 2.4Hz, 1H), 3.52 (d, *J* = 2.5 Hz, 1H). ^13^C NMR (101 MHz, DMSO-*d*_6_) δ 158.58 (d, *J* = 249.0 Hz), 149.05, 141.55, 135.32, 132.01, 131.08 (d, *J* = 8.0 Hz), 130.87, 126.79, 126.01 (d, *J* = 3.9 Hz), 125.86, 124.15, 117.97, 117.32 (d, *J* = 9.7 Hz), 117.08, 87.28. HRMS (ESI) *m/z*: calc. for C_16_H_14_FN_2_O^+^ [M + H]^+^: 289.0538, found 289.0552.

##### 6-Chloro-3-(3-chlorophenyl)-4-methylene-3,4-dihydroquinazolin-2(1H)-one (7u)

White powder. m.p. 245.6~247.2°C;^1^H NMR (400 MHz, DMSO-*d*_6_) δ 10.53 (s, 1H), 7.78 (d, *J* = 2.3 Hz, 1H), 7.61–7.48 (m, 2H), 7.45 (t, *J* = 1.9 Hz, 1H), 7.38 (dd, *J* = 8.6, 2.3 Hz, 1H), 7.29 (dt, *J* = 7.6, 1.7 Hz, 1H), 6.96 (d, *J* = 8.6 Hz, 1H), 4.94 (d, *J* = 2.4 Hz, 1H), 3.47 (d, *J* = 2.5 Hz, 1H). ^13^C NMR (101 MHz, DMSO-*d*_6_) δ 149.28, 142.39, 140.29, 135.23, 134.14, 131.78, 130.75, 130.04, 128.82, 128.76, 126.69, 124.10, 118.31, 117.10, 88.14. HRMS (ESI) *m/z*: calc. for C_15_H_10_Cl_2_N_2_O [M + H]^+^: 305.0243, found 305.0229.

##### 6-Chloro-4-methylene-3-(m-tolyl)-3,4-dihydroquinazolin-2(1H)-one (7v)

White powder. m.p. 201.7~202.4°C; ^1^H NMR (400 MHz, DMSO-*d*_6_) δ 10.45 (s, 1H), 7.74 (d, *J* = 2.3 Hz, 1H), 7.45–7.32 (m, 2H), 7.24 (d, *J* = 7.7 Hz, 1H), 7.11–7.02 (m, 2H), 6.96 (d, *J* = 8.6 Hz, 1H), 4.88 (d, *J* = 2.2 Hz, 1H), 3.49 (d, *J* = 2.2 Hz, 1H), 2.35 (s, 3H). ^13^C NMR (101 MHz, DMSO-*d*_6_) δ 149.44, 142.53, 139.70, 138.79, 135.35, 130.63, 130.08, 129.97, 129.17, 126.63, 126.57, 124.03, 118.34, 117.02, 87.93, 21.23. HRMS (ESI) *m/z*: calc. for C_16_H_13_ClN_2_O [M + H]^+^: 287.0795, found 287.0802.

##### 6-Chloro-4-methylene-3-(o-tolyl)-3,4-dihydroquinazolin-2(1H)-one (7w)

White powder. m.p. 221.2~222.9°C; ^1^H NMR (400 MHz, DMSO-*d*_6_) δ 10.45 (s, 1H), 7.79 (d, *J* = 2.3 Hz, 1H), 7.42–7.32 (m, 5H), 7.20–7.15 (m, 1H), 6.96 (d, *J* = 8.6 Hz, 1H), 4.88 (d, *J* = 2.1 Hz, 1H), 3.36 (d, *J* = 2.2 Hz, 2H), 2.10 (s, 3H). ^13^C NMR (101 MHz, DMSO-*d*_6_) δ 148.99, 141.27, 137.48, 136.50, 135.39, 131.55, 130.75, 129.71, 128.89, 127.95, 126.65, 124.17, 118.06, 117.10, 86.86, 17.15. HRMS (ESI) *m/z*: calc. for C_16_H_13_FN_2_O [M + H]^+^: 285.0789, found 285.0762.

##### 3-(3-Chlorophenyl)-7-methyl-4-methylene-3,4-dihydroquinazolin-2(1H)-one(7x)

White powder. m.p. 214.8~215.9°C; ^1^H NMR (400 MHz, DMSO-*d*_6_) δ 10.34 (s, 1H), 7.60–7.48 (m, 3H), 7.45–7.39 (m, 1H), 7.27 (dt, *J* = 7.7, 1.7 Hz, 1H), 6.84 (dd, *J* = 8.2, 1.7 Hz, 1H), 6.75 (s, 1H), 4.74 (d, *J* = 2.0 Hz, 1H), 3.35 (d, *J* = 2.0 Hz, 1H), 2.28 (s, 3H). ^13^C NMR (101 MHz, DMSO-*d*_6_) δ 149.66, 143.54, 140.75, 140.54, 136.14, 134.08, 131.70, 130.11, 128.90, 128.63, 124.53, 123.75, 115.28, 114.02, 85.57, 21.38. HRMS (ESI) *m/z*: calc. for C_16_H_13_ClN_2_O [M + H]^+^: 285.0789, found 285.0762.

##### 3-(2-Fluorophenyl)-7-methyl-4-methylene-3,4-dihydroquinazolin-2(1H)-one (7y)

White powder. m.p. 224.3~224.9°C; ^1^H NMR (400 MHz, DMSO-*d*_6_) δ 10.44 (s, 1H), 7.57 (d, *J* = 8.1 Hz, 1H), 7.55–7.48 (m, 1H), 7.45–7.39 (m, 2H), 7.35 (ddd, *J* = 7.9, 7.2, 1.4 Hz, 1H), 4.75 (d, *J* = 2.1 Hz, 1H), 3.43 (d, *J* = 2.1 Hz, 1H), 2.08 (s, 2H). ^13^C NMR (101 MHz, DMSO-*d*_6_) δ 156.56 (d, *J* = 247 Hz), 147.29, 140.58, 138.80, 133.98, 129.98, 128.81 (d, *J* = 8.0 Hz), 124.08 (d, *J* = 13.3 Hz), 123.80 (d, *J* = 3.5 Hz), 122.48, 121.78, 115.02 (d, *J* = 19.7 Hz), 113.29, 111.62, 82.67, 19.26. HRMS (ESI) *m/z*: calc. for C_16_H_13_FN_2_O [M + H]^+^: 269.1085, found 269.1062.

##### 7-Methyl-4-methylene-3-(m-tolyl)-3,4-dihydroquinazolin-2(1H)-one (7z)

Blackish green powder. m.p. 161.3~163.6°C; ^1^H NMR (400 MHz, DMSO-*d*_6_) δ 10.25 (s, 1H), 7.54 (d, *J* = 8.1 Hz, 1H), 7.40 (t, *J* = 7.7 Hz, 1H), 7.23 (d, *J* = 7.7 Hz, 1H), 7.09–7.01 (m, 2H), 6.82 (dd, *J* = 8.2, 1.7 Hz, 1H), 6.74 (s, 1H), 4.69 (d, *J* = 1.7 Hz, 1H), 3.36 (d, *J* = 1.7 Hz, 1H), 2.35 (s, 3H), 2.28 (s, 3H). ^13^C NMR (101 MHz, DMSO-*d*_6_) δ 149.82, 143.69, 140.61, 139.60, 139.05, 136.26, 130.18, 129.89, 129.04, 126.73, 124.48, 123.64, 115.19, 114.07, 85.39, 21.37, 21.22. HRMS (ESI) *m/z*: calc. for C_17_H_16_N_2_O [M + H]^+^: 265.1335, found 265.1358.

##### 3-Benzyl-7-methyl-4-methylene-3,4-dihydroquinazolin-2(1H)-one (7aa)

White powder. m.p. 236.8~238.4°C; ^1^H NMR (400 MHz, DMSO-*d*_6_) δ 10.28 (s, 1H), 7.50 (d, *J* = 8.2 Hz, 1H), 7.32 (t, *J* = 7.5 Hz, 2H), 7.28–7.19 (m, 3H), 6.79 (dd, *J* = 8.2, 1.7 Hz, 1H), 6.75 (s, 1H), 4.99 (s, 2H), 4.74 (d, *J* = 2.3 Hz, 1H), 4.06 (d, *J* = 2.4 Hz, 1H), 2.26 (s, 3H). ^13^C NMR (101 MHz, DMSO-*d*_6_) δ 150.72, 140.41, 140.22, 137.58, 135.99, 128.91, 127.18, 126.78, 124.36, 123.68, 115.12, 113.94, 84.68, 46.23, 21.33. HRMS (ESI) *m/z*: calc. for C_17_H_16_N_2_O [M + H]^+^: 265.1335, found 265.1326.

##### 3-Benzyl-6-chloro-4-methylene-3,4-dihydroquinazolin-2(1H)-one (7ab)

White powder. m.p. 237.2~238.9°C; ^1^H NMR (400 MHz, DMSO-*d*_6_) δ 10.44 (s, 1H), 7.71 (d, *J* = 2.2 Hz, 1H), 7.33 (dd, *J* = 8.8, 6.8 Hz, 3H), 7.27–7.21 (m, 3H), 6.95 (d, *J* = 8.6 Hz, 1H), 4.99 (s, 2H), 4.93 (d, *J* = 2.8 Hz, 1H), 4.17 (d, *J* = 2.8 Hz, 1H). ^13^C NMR (101 MHz, DMSO-*d*_6_) δ 150.34, 139.05, 137.27, 135.09, 130.48, 128.95, 127.27, 126.82, 126.62, 123.93, 118.24, 116.97, 87.35, 46.33. HRMS (ESI) *m/z*: calc. for C_16_H_13_ClN_2_O [M + H]^+^: 285.0789, found 285.0776.

## Data Availability

All datasets generated for this study are included in the manuscript/Supplementary Files.

## Author Contributions

All authors listed have made a substantial, direct and intellectual contribution to the work, and approved it for publication.

### Conflict of Interest Statement

The authors declare that the research was conducted in the absence of any commercial or financial relationships that could be construed as a potential conflict of interest.

## Supplementary Material

Supplementary data (^1^H, ^13^C NMR spectrums, computational data, Cartesian coordinates, Crystal Data) associated with this article can be found in the Supporting Information.
