# Supplementary material for: A Simple Metal-Free Cyclization for the Synthesis of 4-Methylene-3-Substituted Quinazolinone and Quinazolinthione Derivatives: Experiment and Theory
Source: Front Chem. 2019 Aug 16;7:584. doi: 10.3389/fchem.2019.00584 (PMC6706782; doi:10.3389/fchem.2019.00584)

checkCIF/PLATON report

Structure factors have been supplied for datablock(s) mo180525d

THIS REPORT IS FOR GUIDANCE ONLY. IF USED AS PART OF A REVIEW PROCEDURE FOR PUBLICATION, IT SHOULD NOT REPLACE THE EXPERTISE OF AN EXPERIENCED CRYSTALLOGRAPHIC REFEREE.

No syntax errors found. CIF dictionary Interpreting this report

Datablock: mo180525d

```
Bond precision:  C-C = 0.0056 A          Wavelength=0.71073

Cell:           a=6.4889(5)    b=10.0104(9)         c=22.6393(19)
                alpha=90       beta=95.7779(14)     gamma=90
Temperature:    296 K

                Calculated                  Reported
Volume          1463.1(2)                   1463.1(2)
Space group     P c                         P c
Hall group      P -2yc                      P -2yc
Moiety formula  C16 H15 N2 O S              ?
Sum formula     C16 H15 N2 O S              C16 H15 N2 O S
Mr              283.36                      283.36
Dx,g cm-3       1.286                       1.286
Z               4                           4
Mu (mm-1)       0.218                       0.218
F000            596.0                       596.0
F000'           596.70
h,k,lmax        8,12,27                     8,12,27
Nref            5722[ 2867]                 5689
Tmin,Tmax       0.915,0.948                 0.893,1.000
Tmin'           0.897

Correction method= # Reported T Limits: Tmin=0.893 Tmax=1.000
AbsCorr = MULTI-SCAN

Data completeness= 1.98/0.99      Theta(max)= 25.999

R(reflections)= 0.0371( 5371)     wR2(reflections)= 0.1027( 5689)

S = 1.071                  Npar= 365
```

The following ALERTS were generated. Each ALERT has the format
test-name_ALERT_alert-type_alert-level.
Click on the hyperlinks for more details of the test.

Alert level C

```
PLAT220_ALERT_2_C Non-Solvent  Resd 1  C   Ueq(max)/Ueq(min) Range        3.7 Ratio
PLAT230_ALERT_2_C Hirshfeld Test Diff for   O1       --C2        .        6.0 s.u.
PLAT230_ALERT_2_C Hirshfeld Test Diff for   C2       --C15       .        6.0 s.u.
PLAT241_ALERT_2_C High   'MainMol' Ueq as Compared to Neighbors of         O1 Check
PLAT242_ALERT_2_C Low    'MainMol' Ueq as Compared to Neighbors of         C2 Check
PLAT340_ALERT_3_C Low Bond Precision on  C-C Bonds ...............    0.00557 Ang.
```

Alert level G

```
PLAT792_ALERT_1_G Model has Chirality at C2        (Polar  SPGR)           S Verify
PLAT792_ALERT_1_G Model has Chirality at C18       (Polar  SPGR)           R Verify
PLAT910_ALERT_3_G Missing # of FCF Reflection(s) Below Theta(Min).         1 Note
PLAT933_ALERT_2_G Number of OMIT Records in Embedded .res File ...         2 Note
PLAT978_ALERT_2_G Number C-C Bonds with Positive Residual Density.         4 Info
```

```
   0 ALERT level A = Most likely a serious problem - resolve or explain
   0 ALERT level B = A potentially serious problem, consider carefully
   6 ALERT level C = Check. Ensure it is not caused by an omission or oversight
   5 ALERT level G = General information/check it is not something unexpected

   2 ALERT type 1 CIF construction/syntax error, inconsistent or missing data
   7 ALERT type 2 Indicator that the structure model may be wrong or deficient
   2 ALERT type 3 Indicator that the structure quality may be low
   0 ALERT type 4 Improvement, methodology, query or suggestion
   0 ALERT type 5 Informative message, check
```

It is advisable to attempt to resolve as many as possible of the alerts in all categories. Often the minor alerts point to easily fixed oversights, errors and omissions in your CIF or refinement strategy, so attention to these fine details can be worthwhile. In order to resolve some of the more serious problems it may be necessary to carry out additional measurements or structure refinements. However, the purpose of your study may justify the reported deviations and the more serious of these should normally be commented upon in the discussion or experimental section of a paper or in the "special_details" fields of the CIF. checkCIF was carefully designed to identify outliers and unusual parameters, but every test has its limitations and alerts that are not important in a particular case may appear. Conversely, the absence of alerts does not guarantee there are no aspects of the results needing attention. It is up to the individual to critically assess their own results and, if necessary, seek expert advice.

Publication of your CIF in IUCr journals

A basic structural check has been run on your CIF. These basic checks will be run on all CIFs submitted for publication in IUCr journals (*Acta Crystallographica*, *Journal of Applied Crystallography*, *Journal of Synchrotron Radiation*); however, if you intend to submit to *Acta Crystallographica Section C* or *E* or *IUCrData*, you should make sure that full publication checks are run on the final version of your CIF prior to submission.

Publication of your CIF in other journals

Please refer to the *Notes for Authors* of the relevant journal for any special instructions relating to CIF submission.

PLATON version of 23/04/2018; check.def file version of 23/04/2018

Datablock mo180525d - ellipsoid plot

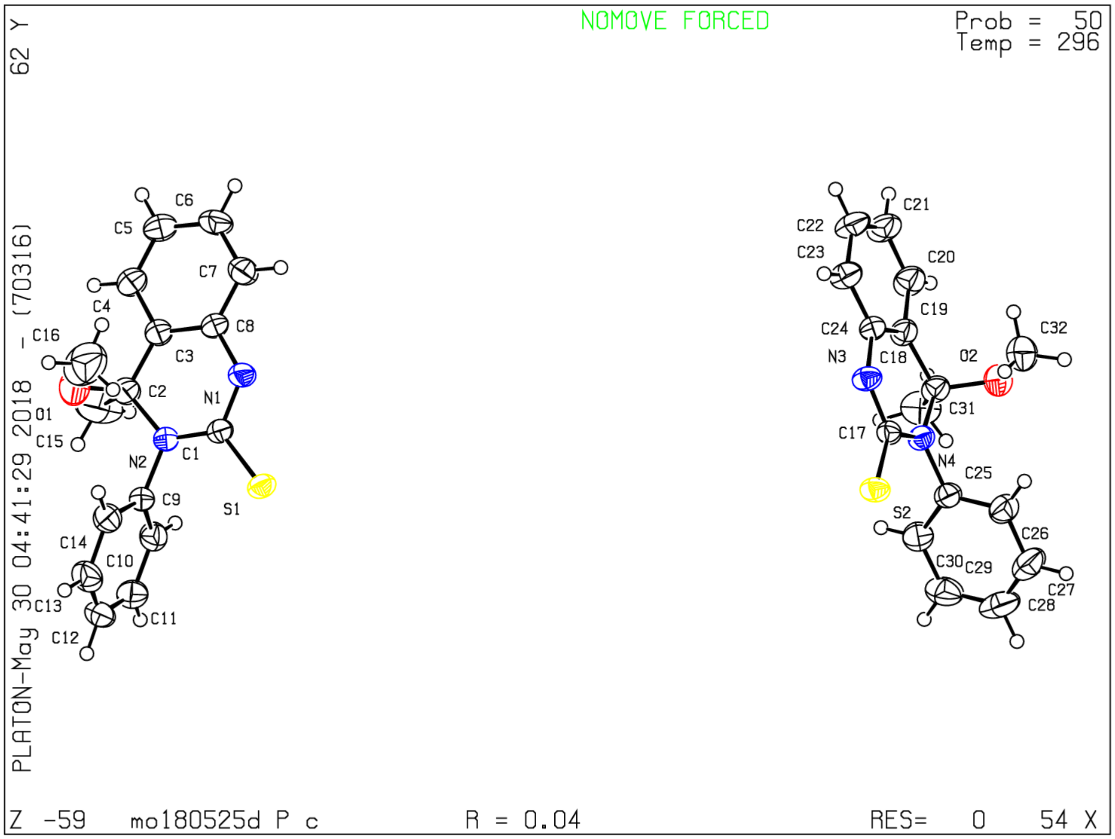

Supplement: Supplementary file 1 [file Data_Sheet_1.ZIP › CDCC1906268 .pdf]
